# K-HEARS SCREEN: ETHNIC CHURCH-BASED HEARING SCREENING FOR KOREAN IMMIGRANT OLDER ADULTS IN THE UNITED STATES

**DOI:** 10.1093/geroni/igae098.0433

**Published:** 2024-12-31

**Authors:** Hae-Ra Han, Joshua Betz, Jacqueline Kwak, Joanne Park, Jami Trumbo, Carrie Nieman

**Affiliations:** Johns Hopkins University, Baltimore, Maryland, United States; Cochlear Center for Hearing & Public Health, Baltimore, Maryland, United States; Johns Hopkins University, Baltimore, Maryland, United States; Johns Hopkins University, Baltimore, Maryland, United States; Johns Hopkins University, Baltimore, Maryland, United States; Johns Hopkins University School of Medicine, Baltimore, Maryland, United States

## Abstract

Age-related hearing loss is highly prevalent in the United States (US), yet there is limited information on the magnitude of hearing loss in certain racial/ethnic minority groups, particularly those with limited English proficiency, due, in large part, to limited accessibility to hearing screening programs and national cohorts of older adults primarily focused on English-proficient communities. Older Korean Americans (KAs)—one of the fastest-growing aging populations in the US—are predominantly monolingual first-generation immigrants. We investigated the prevalence of hearing loss in community-dwelling KA older adults (60+ years) residing in the Baltimore-Washington metropolitan area. Faith-based organizations, such as ethnic churches, serve as epicenters for health promotion activities across diverse immigrant communities. To this end, we mobilized 18 Korean ethnic churches in the target area and screened 514 older KAs (mean=72.6 years, 61.5% women, 54.1% <$50K annual household income, and 97.8% limited English). We found that more than half (55.4% or n=285) had a clinically significant hearing loss and 56% of individuals reported never having their hearing screened. Of those with hearing loss, 61% had a mild degree of hearing loss and 39% with moderate or greater degree of hearing loss. Despite the high rates of hearing loss, prevalence of current hearing aid use was low (12%), similar to findings in other under-resourced communities. To the authors’ knowledge, these findings represent the first estimates regarding hearing health among older KAs. Our findings suggest the urgent need for addressing hearing care disparities in this linguistically and socially disadvantaged group of immigrant older adults.

